# Neutrophil extracellular traps and parasite load are associated with dermal matrix remodeling in Leishmania braziliensis infection

**DOI:** 10.1590/0037-8682-0506-2025

**Published:** 2026-07-03

**Authors:** Gislainy Pereira da Silva, Joselina Maria da Silva, Diego Luiz Doneda, Eudes Thiago Pereira Ávila, Vanina Danuza Toso, Marcia Hueb, Thiago da Rosa Lima, Amílcar Sabino Damazo

**Affiliations:** 1 Universidade Federal de Mato Grosso, Programa de Pós-Graduação em Ciências da Saúde, Cuiabá, MT, Brasil.; 2 Universidade Federal de Mato Grosso, Departamento de Medicina Clínica, Cuiabá, MT, Brasil.; 3 Universidade Federal de Mato Grosso, Departamento de Química, Cuiabá, MT, Brasil.; 4 Universidade de Brasília, Programa de Pós-Graduação em Medicina Tropical, Brasília, DF, Brasil.

**Keywords:** Cutaneous leishmaniasis, Neutrophils, Leishmania braziliensis, Amastigotes, Extracellular matrix

## Abstract

**Background:**

Cutaneous leishmaniasis is a chronic parasitic condition. The association of neutrophil extracellular traps with dermal extracellular matrix degradation, parasite load, and leukocyte profiles in cutaneous leishmaniasis lesions remains underexplored. This study aimed to investigate these associations in patients with cutaneous leishmaniasis.

**Methods:**

Patients (n=78) from Cuiabá, Mato Grosso, Brazil, diagnosed with cutaneous leishmaniasis were included. Biopsy samples were collected.. Species were identified by polymerase chain reaction. Leukocytes, amastigotes, and neutrophil extracellular traps were identified and quantified, and extracellular matrix degradation was assessed using immunohistochemistry, Masson’s trichrome, Sirius Red, and reticulin stains. Statistical associations were determined using Pearson’s correlation coefficient.

**Results:**

All patients exhibited L. braziliensis infection. Most patients (76.9%) had cellular and exudative reaction lesions, while 23.1% exhibited granulomatous and exudative reaction lesions. A significant correlation was observed between extracellular matrix degradation and amastigote load in cellular and exudative reaction lesions (r=0.599; p=0.0001) but not in granulomatous and exudative reaction lesions (r=0.068; p=0.263). Similarly, in cellular and exudative reaction lesions, neutrophil extracellular trap formation showed a strong positive correlation with amastigote numbers (r=0.423; p=0.0001), a relationship absent in granulomatous and exudative reaction lesions (r=0.0009; p=0.9036).

**Conclusion:**

In L. braziliensis infection with cellular and exudative reaction lesions, neutrophil extracellular traps function as an active, proportional defense mechanism against parasite load. However, in granulomatous and exudative reaction lesions, they contribute to a more structured containment but lack a direct proportional relationship to parasite numbers. This distinction provides critical insights into cutaneous leishmaniasis pathogenesis.

## INTRODUCTION

The immunological response to Leishmania braziliensis is a complex process in which the host’s defense mechanisms often contribute to the clinical manifestation of the disease. This reaction involves several cellular and molecular stages, including immune activation and cellular response, cytokine production, and reactive oxygen species^1^ ^-^ ^13^. These changes, particularly the oxidative state of the infected site, can interfere with wound healing and impair tissue regeneration^6^. Anti-leishmanial treatment aims to reduce both the duration and severity of the disease, as well as scar formation, with the most common therapies being pentavalent antimonials and pentamidine^7^.

Several infectious agents can invade and multiply within human cells. Protozoa of the genus Leishmania are intracellular parasites of the human mononuclear phagocyte system^2^; however, these parasites have also been detected within neutrophils^8^. Neutrophils are the first cells recruited to the site of infection in leishmaniasis, and their role in controlling parasite load has been demonstrated in experimental models^2^ ^,^ ^8^ ^,^ ^10^ ^,^ ^11^ ^,^ ^13^. However, some researchers have suggested that neutrophils play a detrimental role in murine leishmaniasis, causing more severe disease associated with a Th2 response^10^, or even serving as a vehicle for Leishmania entry into macrophages^11^ ^,^ ^12^. Neutrophils release a meshwork of chromatin associated with granular and cytoplasmic proteins, known as neutrophil extracellular traps (NETs)^13^ ^-^ ^15^.

NETs can trap promastigotes and amastigotes of various Leishmania species^11^ ^,^ ^16^ ^-^ ^17^. NET formation is dependent on chromatin release and the activities of elastase, myeloperoxidase, and peptidylarginine deiminase 4^13^ ^,^ ^14^ ^,^ ^18^ ^-^ ^20^. NET formation has been reported in studies involving distinct species, such as L. amazonensis, L. chagasi, L. donovani, L. major and L. infantum^21^ ^-^ ^23^, L. guyanensis^24^, L. braziliensis^15^, and L. panamensis^25^.

Owing to the enzymatic activity of NETs, the extracellular matrix (ECM) undergoes degradation, which impedes parasite migration in the dermis of patients with cutaneous leishmaniasis (CL)^26^. In vitro studies have demonstrated the ability of promastigotes to bind to and move through type I collagen fibers^27^. Parasite invasion of type I collagen fibers leads to their remodeling and degradation, possibly mediated by metalloproteinases and cysteine proteinases^28^. During the chronic phase of infection, type I collagen is replaced by type III collagen^29^. A higher content of type III collagen during leishmaniasis can result in a softer dermal matrix, through which parasites may have an easier path for migration and tissue invasion^30^. 

This study aimed to evaluate the association between dermal ECM degradation in patients with CL and the presence of amastigotes, leukocytes, and NETs.

## METHODS

### Patients

This study was conducted at the Hospital Universitário Júlio Müller (HUJM) of the Federal University of Mato Grosso in Cuiabá, Mato Grosso, Brazil. We enrolled 78 patients diagnosed with CL from this clinic, which serves as a state-level reference for leishmaniasis care. The center routinely manages patients from across the entire state of Mato Grosso and neighboring regions in the North and Central-West of the country. The mean age of the patients was 55 years (range, 18-56 years). Patients with CL were considered eligible for this study if they had no other infectious or chronic degenerative diseases, did not present with an immunosuppressive condition, and had not yet initiated treatment for CL. The study was conducted in 2024. The size of the target population was obtained from official surveillance records provided by the State Health Secretariat of Mato Grosso. The required sample size was estimated assuming an 80% confidence level and a 5% margin of error, based on the total number of reported CL cases in Cuiabá, Mato Grosso, Brazil, during the study year.

Personal data, including age, sex, residence, and clinical history (origin and symptom onset), were documented for all participants. The HUJM medical staff conducted comprehensive physical evaluations, specifically examining the general health of the patients alongside a detailed assessment of lesion morphology, including the size, location, and anatomical sites.

### Ethical Considerations

This study was conducted in strict adherence to the ethical principles outlined in the Declaration of Helsinki (1964 and its subsequent revisions through 2000). Before data collection, all participants were informed of their right to decline participation without prejudice. Written informed consent was obtained from each patient following approval by the HUJM Ethics Review Board (Approval No. 1.653.110). Furthermore, the use of biopsy material collected during routine clinical examinations ensured that no additional risks were posed to the study population.

### Laboratory Examinations

To obtain a positive diagnosis for CL, a biopsy sample was collected for histopathological analysis, and a cervical brush was used to collect a sample from the lesion border for polymerase chain reaction (PCR).

Local antisepsis was achieved by applying a povidone-iodine solution and 0.9% saline to the target area. Once the site was cleaned, 2% lidocaine was administered as an infiltrative anesthetic. Subsequently, a biopsy was performed using a 4 mm punch tool.

### Sample Collection with a Cervical Brush, DNA Extraction, and Identification of Leishmania Species in Patients via PCR-HSP70C

Samples were collected using cervical brushes in direct contact with the lesions. After collection, the brush was stored in a 1.5 mL microtube containing phosphate-buffered saline (PBS).

Genomic DNA was isolated from the cervical brushes using Invitrogen® (USA) commercial kits following the manufacturer’s instructions. This procedure integrated an initial digestion phase utilizing proteinase K. To determine the concentration of the resulting genetic material, a Nanodrop® spectrophotometer was used for DNA quantification. Leishmania species were identified via the PCR-HSP70C^3^ method. Each 30 µL reaction mixture comprised 6.2 µL of template DNA, 0.3 µL of GoTaq® DNA polymerase, and 0.6 µL each of forward (5'-GGACGAGATCGAGCGCATGGT-3') and reverse (5'-TCCTTCGACGCCTCCTGGTTG-3') primers. Additionally, the solution contained 3 µL of buffer, 3 µL of dNTPs, 1.8 µL of 1.5 mM MgCl₂, and 14.5 µL of ultrapure water. The thermal cycling conditions for the PCR assays involved an initial denaturation at 94°C for 4 min, followed by 33 cycles of denaturation (94°C for 15 s), annealing (58°C for 45 s), and extension (72°C for 30 s), with a concluding extension at 72°C for 10 min. To confirm amplification, the PCR-HSP70C products were analyzed using 2% agarose gel electrophoresis to visualize characteristic bands.

For cases where Leishmania DNA was detected, species identification was conducted using restriction fragment length polymorphism. The HSP70C amplification products were subjected to enzymatic digestion by incubation with HaeIII and BstUI (separately) for 12 h at 37°C. Each 20 µL digestion reaction contained 8 µL of PCR product, 9 µL of ultrapure water, 2 µL of buffer, and 1 µL of the respective enzyme.

### Histopathological Analysis

Tissue biopsies were fixed in PBS-buffered 4% paraformaldehyde and transported to the laboratory for histological preparation. The samples were cleared in xylene after graded ethanol dehydration and subsequently embedded in paraffin blocks. A HIRAX M60 microtome was used to obtain 3-µm sections, which were stained with hematoxylin and eosin following standard deparaffinization protocols. Finally, two specialist researchers independently performed a morphological classification of CL lesion types, categorizing the histopathological patterns in accordance with the description by de Magalhães et al.³¹: cellular and exudative reaction (CER); necrotic and exudative reaction; necrotic-granulomatous and exudative reaction; granulomatous and exudative reaction (GER); and tuberculoid and exudative reaction.

Masson’s trichrome staining was used to evaluate the ECM; the intact matrix was stained blue, while the degraded matrix was stained red. Picrosirius red staining was performed under polarized light to evaluate type I collagen, while reticulin staining was performed to evaluate type III collagen.

Slides containing histological sections were analyzed by two distinct blinded evaluators under an Axioscope A1 light microscope (Zeiss, Germany), and images were captured using AxioVision software (Zeiss, Germany).

### Immunohistochemical Evaluation

Amastigotes and NETs were detected by immunohistochemistry. Leishmania amastigote detection was performed as described in a previous study^32^ with an antibody against oligopeptidase B^33^. For this analysis, 3-µm-thick sections were obtained and adhered to silanized slides. The analysis was conducted using a light microscope, examining five microscopic fields. The histological sections were deparaffinized in an oven at 60°C, followed by tissue antigen retrieval using a 0.4% sodium citrate solution in a water bath for 20 min. Next, the slides were washed in Tris-buffered saline (pH, 8.0) at room temperature. This was followed by blocking endogenous peroxidase with hydrogen peroxide and protein blocking with 10% goat serum for 5 min each. The primary antibodies used were the mouse anti-oligopeptidase B of Leishmania (1:200 in PBS) and the rabbit anti-elastase (1:200 in PBS) (Thermofisher, USA). The reaction was visualized using the Easy-link DUO system kit (Easy Path, USA), which utilizes an horseradish peroxidase (HRP) polymer reaction system and a DAB (3,3′-Diaminobenzidine) solution for amplification, according to the manufacturer’s specifications.

### Image Analysis of Inflammatory cells and Amastigotes

We quantified the presence of amastigotes and inflammatory cells (plasma cells, lymphocytes, macrophages, and neutrophils) using ImageJ 1.54 (U.S. National Institutes of Health, USA). Data were obtained from 900 × 300 pixel regions of interest (ROIs) captured within both the deep and papillary dermis. The results were normalized and expressed as the average count per mm², with each evaluated field covering a specific area of 0.015 mm².

### Evaluation of Collagenesis and NETs

Collagenesis and NETs, observed in the slides stained with Masson’s trichrome and by immunohistochemistry, were measured as follows: Six different fields were photographed at 100× magnification (three fields from the upper epidermis-dermis and three fields from the lower dermis-hypodermis), and an ROI of 320 × 40 pixels (= 2.62 × 0.33 μm, converted using ImageJ) was selected for each field. The “color deconvolution” plugin of the ImageJ software was used to assess the percentage of blue staining (collagen) and brown staining (NETs, HRP-DAB immunohistochemistry) in the image area. The staining method was selected, and the software recognized the colors in the image and decomposed them into three basic colors. For Masson’s trichrome staining, the image was decomposed into blue (collagen), red, and purple. The distribution of the blue color (collagen) was quantified as the mean distribution of the collagen area per treatment using the “Threshold” plugin of the ImageJ software. The same procedure was performed on the slides stained with immunohistochemistry for NETs, picrosirius red for type I collagen, and reticulin for type III collagen.

### Statistical Analysis

Statistical analyses were conducted using the SPSS software (version 25). For all analyses, data are expressed as the mean ± standard deviation. The time of lesion onset before treatment was evaluated using the Mann-Whitney test. The correlation between injury description and histopathological lesion was determined using the chi-square test. The association between ECM degradation data and the findings from histopathological analysis, leukocyte identification, NETs, and amastigotes was evaluated. This association was determined using Spearman’s correlation coefficient. P-values < 0.05 were considered statistically significant.

## RESULTS

### Clinical and Histopathological Data

All patients included in the study tested positive for L. braziliensis infection (Supplementary Material Figure 1). The cohort consisted of 51 men (65.4%) and 27 women (34.6%). Regarding self-reported skin color, five patients were classified as white (6.4%), and 73 as brown-skinned (93.6%). Lesion duration ranged from 1 to 7 months. The most common lesion types were ulcerated (22%), infiltrated (14%), granulomatous and infiltrated (14%), and granulomatous (14%) lesions. Most patients had more than one lesion, and the most frequent region was the lower limbs (62%) (Table 1).

**TABLE 1:** Epidemiological and clinical data of patients with cutaneous leishmaniasis.

Variables analyzed		Number (n)	Percentage (%)
Sex (n=78)	Male	51	65.4
	Female	27	34.6
Skin color (n=78)	White	5	6.4
	Brown	73	93.6
Injury description (n=126)	Ulcerated with raised edges, granular bottom	10	7.9
	Ulcerated and infiltrated	10	7.9
	Ulcerated without infiltrated border	10	7.9
	Granulomatous	17	13.5
	Ulcerated, infiltrated, raised edge, granulomatous background	10	7.9
	Infiltrated	17	13.5
	Ulcerated	28	22,2
	Bleeding	7	5,7
	Granulomatous and infiltrated	17	13.5
Injury site (n=93)	Face	6	6.5
	Trunk	4	4.3
	Upper limb	24	25.8
	Lower limb	59	63.4

Histopathological analysis classified lesions into two main types. Most patients exhibited CER-type lesions (n=60; 76.9%), while a smaller proportion presented with GER-type lesions (n=18; 23.1%). The average lesion duration showed no statistically significant difference between the two groups (CER, 161.4 ± 118.8 days; GER, 195.3 ± 173.2 days; p=0.45). No correlation was observed between the histopathological and anatomical types of lesions (Supplementary Material Table 1).

### Analysis of Collagen Fibers

**FIGURE 1:**
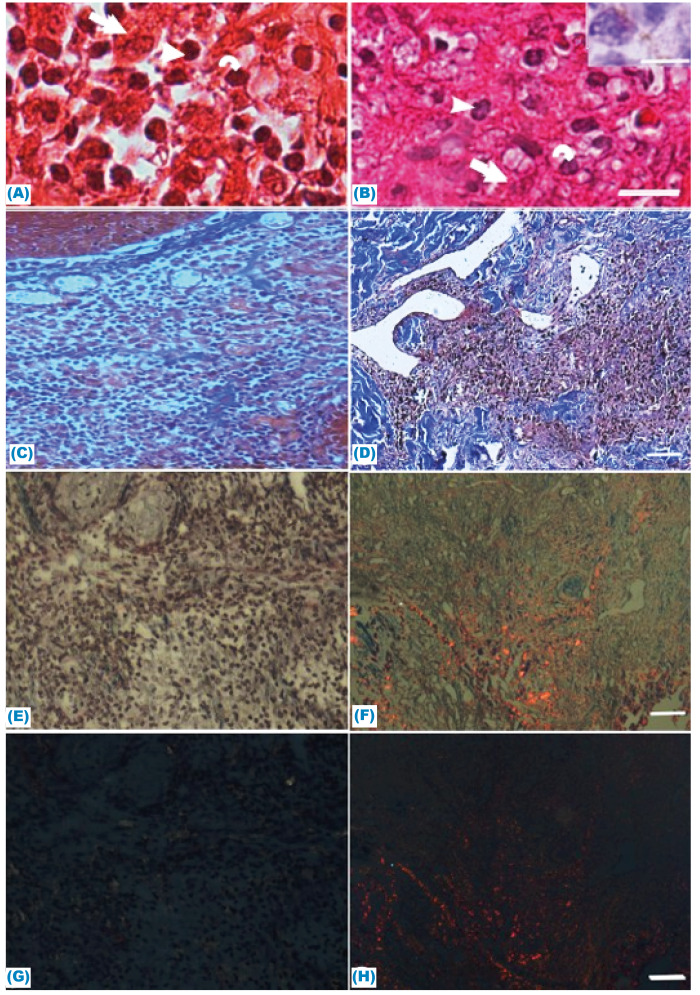
Histochemical analysis of cutaneous leishmaniasis lesions. (A) Analysis of a cellular and exudative reaction (CER)-type lesion showing macrophages (arrow), neutrophils (arrowhead) and lymphocytes (curved arrow). (B) The granulomatous and exudative reaction (GER)-type lesion showed histolymphoplasmacytic infiltrates in the granuloma. Macrophages (arrow), neutrophils (arrowhead) and lymphocytes (curved arrow). Insert: Immunohistochemical staining amastigotes. (C) Analysis of a CER-type lesion showing degraded extracellular matrix stained in red. (D) The GER-type lesion showed that the intact matrix around the granuloma is stained blue. (E) Identification of type III collagen fibers in a CER-type lesion. (F) Identification of type III collagen fibers in a GER-type lesion. (G) CER-type lesion showing the absence of type I collagen fibers. (H) GER-type lesion showing sparse type I collagen fibers visualized by polarization. Staining: Hematoxylin and eosin (A-B); Masson’s trichrome (C-D); Reticulin (E-F); Sirius red (G-H); Immunohistochemistry (Insert). Scale bars: 10 μm (A, B and insert) and 50 μm (C-F).

In patients with CER-type lesions, an intense inflammatory infiltrate was present in the papillary and reticular dermis. The infected skin displayed alterations in the dermal matrix, characterized by collagen degradation, neutrophils, and macrophages (Figure 1A and Figure 1B). Amastigotes were detected in the cytoplasm of macrophages and in the ECM (Figure 1 insert). Masson’s trichrome staining revealed an absence of the collagen matrix that supports inflammatory cells within the dermis (Figure 1C). In these CER lesions, 34.3 ± 17.7% of the ECM was stained by Masson’s trichrome, indicating the portion of matrix that was not degraded by the inflammatory process. In GER-type lesions, analyses showed an intense inflammatory infiltration within a granuloma composed primarily of parasitized macrophages and lymphocytes. Masson’s trichrome staining demonstrated that 32.7 ± 13.2% of the matrix remained non-degraded in these lesions (Figure 1D).

Furthermore, reticulin staining revealed the presence of type III collagen adjacent to the inflammatory process (Figures 1E and 1F), whereas picrosirius red staining indicated an absence of type I collagen (Figures 1G and 1H). Reticulin evidenced that GER-type lesions increased in type III collagen associated with the inflammatory cells (Figure 1F). Additionally, picrosirius red also showed an intense alteration of type I collagen within the granuloma’s ECM (Figure 1H). Neoformation of both type I and type III collagen was observed in the peripheral dermis, adjacent to the granuloma (Figure 1F and 1H ).

**FIGURE 2:**
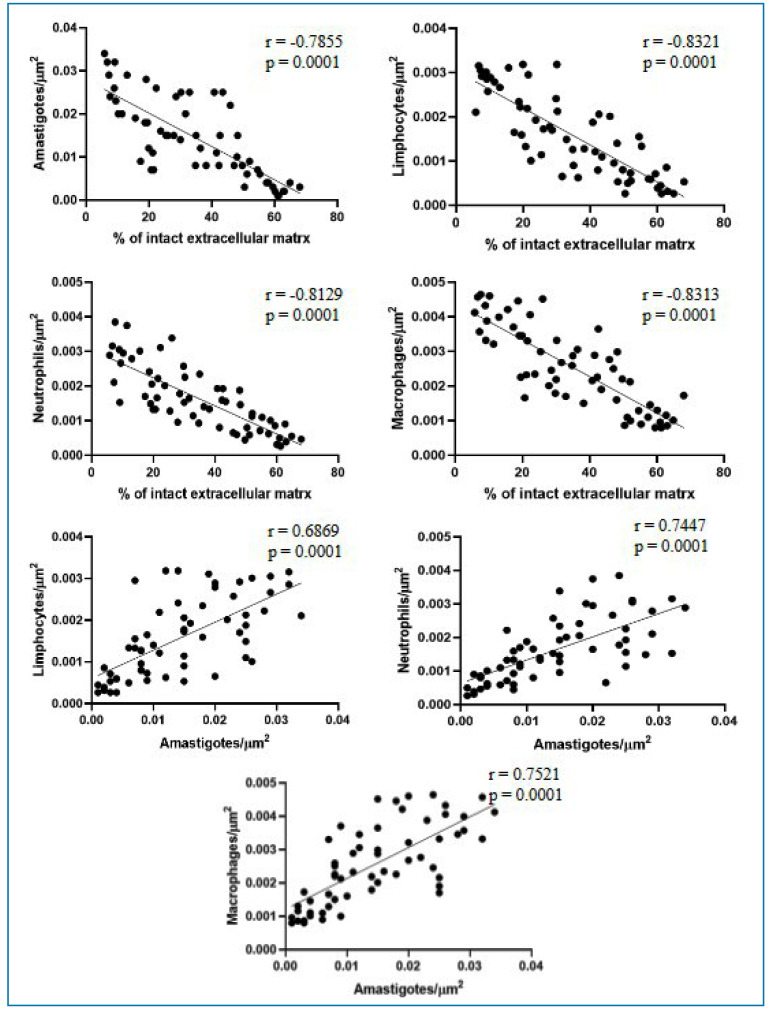
Correlation analysis of the extracellular matrix with the number of parasites and number of leukocytes in cellular and exudative reaction-type lesions from patients with cutaneous leishmaniasis. The number of patients was 60. Statistical analyses were performed using Spearman’s correlation.

In the histopathological evaluation of CER lesions, Masson’s trichrome staining demonstrated discrete ECM fiber degradation in patients with a low amastigote burden. In contrast, in patients with a high amastigote burden, the matrix change was more evident, culminating in a substantial loss of normal dermal architecture (r=-0.7855; p=0.0001) (Figure 2). 

**FIGURE 3:**
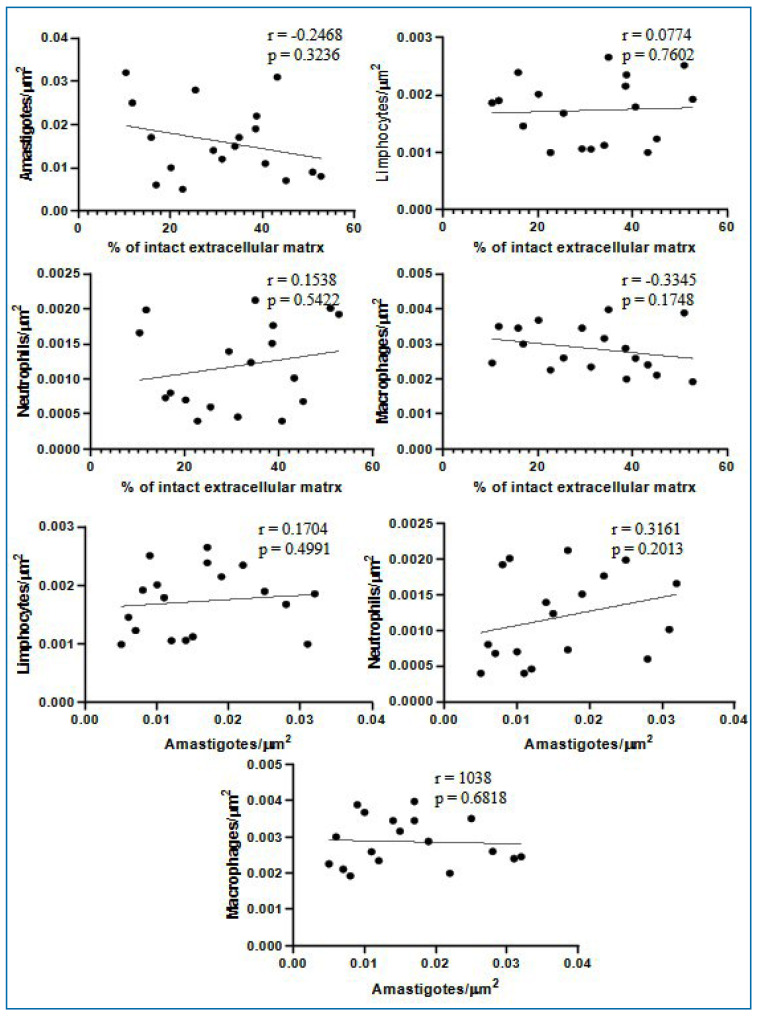
Correlation analysis of the extracellular matrix with the number of parasites and number of leukocytes in granulomatous and exudative reaction-type lesions from patients with cutaneous leishmaniasis. The number of patients was 18. Statistical analyses were performed using Spearman’s correlation.

A negative correlation was observed between matrix degradation and the number of leukocytes, including lymphocytes (r=-0.8321; p=0.0001), neutrophils (r=-0.8129; p=0.0001), and macrophages (r=-0.8313; p=0.0001) (Figure 2). However, when evaluating the same data in GER-type lesions, no such correlation was found between the percentage of matrix degradation and the number of amastigotes, lymphocytes, neutrophils, or macrophages (Figure 3).

### Analysis of Leukocytes and Amastigotes according to the Skin Lesion Type

In CER-type lesions, leukocytes were heterogeneously distributed throughout the inflammatory infiltration. A negative correlation was found between the number of amastigotes and the leukocyte infiltrate, specifically with lymphocytes (r=0.6869; p=0.0001), neutrophils (r=0.7447; p=0.0001), and macrophages (r=0.7521; p=0.0001) (Figure 2). In contrast, the same analysis revealed no correlation between the cellular infiltrate and amastigote numbers in GER-type lesions (Figure 3). 

### Analysis of NETs and Amastigotes according to the Skin Lesion Type

**FIGURE 4:**
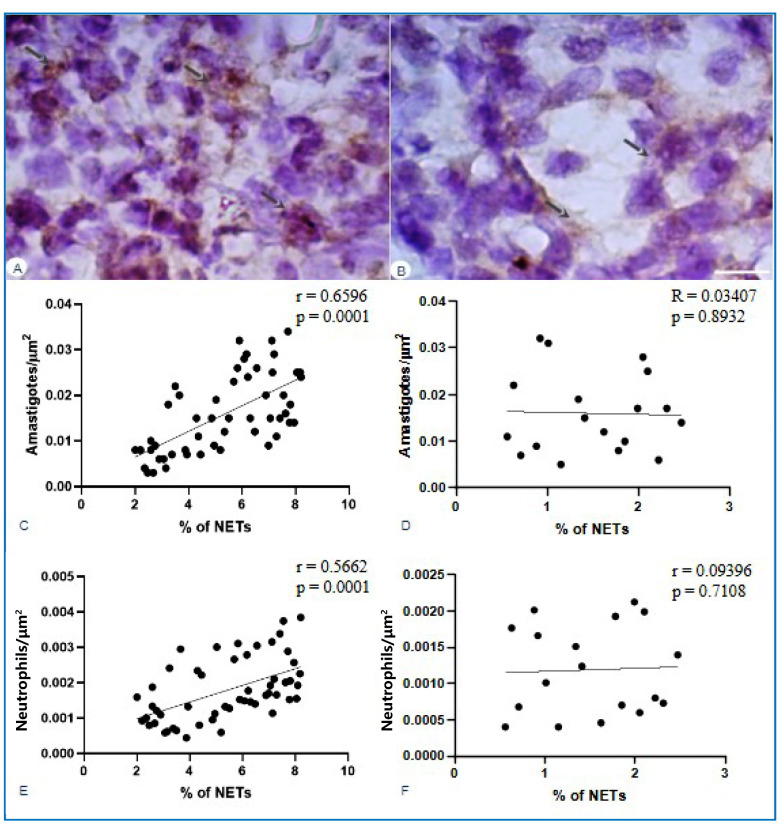
Immunohistochemical analysis of cutaneous leishmaniasis lesions. (A and B) cellular and exudative reaction (CER)-type and granulomatous and exudative reaction (GER)-type lesions showing the staining of NETs in the dermis (arrows). Counterstain: Hematoxylin. Scale bars: 10 μm. Correlation analysis of the percentage of NETs with the number of parasites (C and D) and number of neutrophils (E and F) in CER- and GER-type lesions from patients with CL, respectively. The number of patients with CER and GER lesions was 60 and 18, respectively. Statistical analyses were performed using Spearman’s correlation.

NET formation was observed in 88.4% of CER-type lesions and in 100% of GER-type lesions. In positive CER cases, the quantity of NETs varied according to the presence of neutrophils and amastigotes (Figure 4). Immunohistochemical staining confirmed the presence of areas occupied by NETs in direct contact with amastigotes (Figures 4A and 4B ). The distribution of NETs showed a positive correlation with the number of amastigotes in CER-type lesions (r=0.6596; p=0.0001), but not in GER-type lesions (Figures 4C and 4D ). Similarly, the number of neutrophils showed a positive correlation with the number of amastigotes in CER-type lesions (r=0.5662; p=0.0001), but not in GER-type lesions (Figures 4E and 4F ). In patients in whom NETs were not identified, the amastigote count was low, ranging from 0.001 to 0.004 amastigotes/µm². 

## DISCUSSION

Parasitemia, leukocyte count, and the cellular mechanisms of parasite capture are key factors for better understanding the dynamics of CL. This study presents data correlating the histopathological process of cutaneous lesions in patients with CL with the presence of leukocytes, amastigotes, and NET formation during L. braziliensis infection.

The epidemiological and clinical data presented in this study outline a profile consistent with what is already known for the state of Mato Grosso. The predominance of male patients (65.4%), mixed ethnicity (93.6%), and the majority of the lesions being present on the lower limbs (62%) is consistent with previous studies conducted in the same region^3^ ^-^ ^5^ ^,^ ^12^. In the histopathological classification of the lesions, most patients (76.9%) presented with CER-type lesions, whereas a smaller proportion (23.1%) exhibited GER-type lesions. These findings validate the clinical and epidemiological relevance of this study. The histopathological heterogeneity between CER and GER lesions generates different infectious microenvironments in which the parasite interacts with the host^5^ ^,^ ^31^, directly influencing tissue remodeling. Silva et al.^5^ described that most patients with CER lesions have early infections and exhibit a stronger cytotoxic immunological response, whereas most patients with GER lesions present an immunological response with M2 macrophages.

Histochemical analysis revealed information that allowed for the evaluation of the extent of ECM degradation caused by the inflammatory process induced by L. braziliensis. On average, patients with CER and GER lesions showed a similar percentage of ECM degradation. In the lesions, the presence of an intense inflammatory infiltration in the reticular dermis was associated with type I collagen degradation. Furthermore, an increase in type III collagen was observed when compared with the skin at the tissue margins. The replacement of type I collagen with type III during the chronic phase of Leishmania infection has been described in other studies^29^. A higher content of type III collagen may result in a more pliable dermis, which can facilitate parasite migration and invasion^30^. The presence of new type I and type III collagen formation in the dermis surrounding the granuloma in GER-type lesions signals an attempted tissue repair process, which coexists with infection-induced ECM degradation and reorganization^34^. Neutrophils release potent enzymes, such as elastase and myeloperoxidase, which can degrade ECM components^13^ ^,^ ^14^. Additionally, the Leishmania parasite produces metalloproteinases and cysteine proteinases capable of invading and degrading collagen fibers^35^ ^-^ ^37^. Therefore, the degradation of type I collagen in CER lesions results from a combined action of enzymes released by the host’s inflammatory cells and the parasite’s metalloproteinases, leading to remodeling of the tissue microenvironment. ECM degradation is not merely a consequence of the infection but an active process that can influence the disease outcome.

Upon more specific evaluation of neutrophils at the site of L. braziliensis infection, the presence of NETs can be identified in the dermis, where they form a scaffold for parasite capture and destruction. The presence of neutrophils and their role in controlling parasite load via NETs has been demonstrated in experimental models^9^ ^,^ ^29^ ^,^ ^17^ and in the cutaneous lesions of patients with L. guyanensis^24^, L. braziliensis^15^, and L. panamensis^25^.

NETs were observed in patients with CER-type lesions with a moderate-to-high parasite load and showed a positive correlation with the number of neutrophils and amastigotes. In addition to contributing to ECM degradation^26^, NETs contain other molecules that aid in macrophage activation, thereby promoting the defense against both promastigotes and amastigotes of various Leishmania species^11^ ^,^ ^16^ ^,^ ^17^. This correlation suggests that in CER lesions, NET production is a direct response, quantitatively linked to the intensity of the infection and cellular recruitment.

However, in GER-type lesions, NETs did not correlate with the number of amastigotes or neutrophils. This lack of correlation may indicate a more complex immune dynamic, which is characteristic of the granulomatous environment. The histopathological heterogeneity between CER and GER lesions generates different infectious microenvironments in which the parasite interacts with the host^5^ ^,^ ^31^, directly influencing tissue remodeling. Immune cells are usually established within granulomas, and the response may be more regulated^38^. Although present in all GER-type lesions, as described in another study^15^, NET production within the granuloma may not be an essential mechanism in the containment effort.

This study has some limitations. The absence of longitudinal follow-up for patients is a significant restriction, as it prevents evaluation of the skin healing process and lesion recurrence over time. Although the number of patients is considerable for a clinical-histopathological study, it is limited in its capacity to represent the entire heterogeneous population affected by CL. Furthermore, the lack of a complete immunological profile assessment for the patients restricts a deeper understanding of the host’s responses, which is a critical factor for an immune-mediated disease such as CL. Such limitations preclude the inference of direct and definitive causality between the findings and disease progression or treatment efficacy.

In conclusion, the data from this study indicate the plasticity of the inflammatory response in CL induced by L. braziliensis. In CER lesions, NETs play an active and directly responsive role in combating parasite load, functioning as a line of defense in conjunction with leukocytes and acting proportionally to the intensity of the infection. In contrast, in GER lesions, NET formation is an integral part of a more structured containment mechanism, in which the numerical relationship between parasites and NETs is not proportional. This distinction further broadens the understanding of CL pathogenesis and may have implications for future therapeutic strategies. 

## Data Availability

Research data is only available upon request.
