# Supplementary material for: Neutrophil extracellular traps and parasite load are associated with dermal matrix remodeling in Leishmania braziliensis infection
Source: Rev Soc Bras Med Trop. 2026 Jul 3;59:e0506-2025. doi: 10.1590/0037-8682-0506-2025 (PMC13331191; doi:10.1590/0037-8682-0506-2025)
Supplement: Supplementary Material Figure 1 [file 1678-9849-rsbmt-59-e0506-2025-md1.pdf]

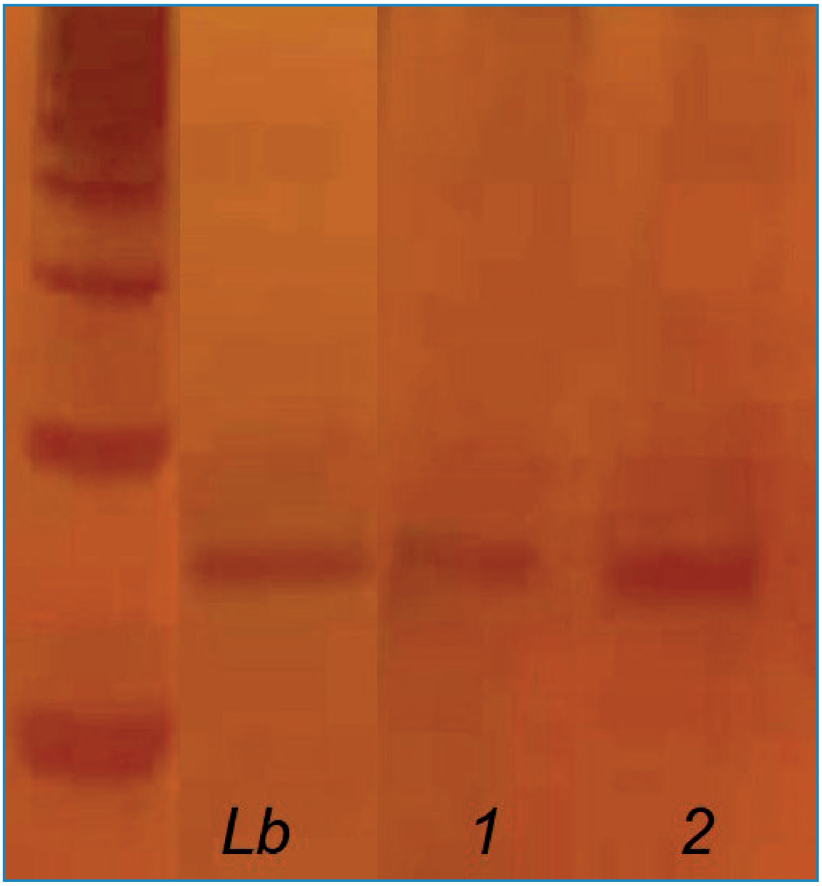

SUPPLEMENTARY MATERIAL FIGURE 1: Identification of Leishmania species by PCR-RFLP. The first column represents the molecular weight marker. After digestion of the products with the BstUI enzyme, the second column represents the pattern for *Leishmania braziliensis* (Lb) species, while the third and fourth columns represent samples from patients with CL (1 and 2).
