# Supplementary material for: Neutrophil extracellular traps and parasite load are associated with dermal matrix remodeling in Leishmania braziliensis infection
Source: Rev Soc Bras Med Trop. 2026 Jul 3;59:e0506-2025. doi: 10.1590/0037-8682-0506-2025 (PMC13331191; doi:10.1590/0037-8682-0506-2025)
Supplement: Supplementary Material Table 1 [file 1678-9849-rsbmt-59-e0506-2025-md2.pdf]

SUPPLEMENTARY MATERIAL TABLE 1: Injury description and histopathological characterization of patients with cutaneous leishmaniasis.

Injury description	Histopathological lesion		p value
	CER	GER	
Ulcerated with raised edges, granular bottom	4	3	0.6590
Ulcerated and infiltrated	6	3	
Ulcerated without infiltrated border	6	2	
Granulomatous	12	2	
Ulcerated, infiltrated, raised edge, granulomatous background	3	2	
Infiltrated	9	1	
Ulcerated	15	3	
Granulomatous and infiltrated	5	2	

P value obtained by chi-square test.
